# Sequence Complexity of Amyloidogenic Regions in Intrinsically Disordered Human Proteins

**DOI:** 10.1371/journal.pone.0089781

**Published:** 2014-03-03

**Authors:** Swagata Das, Uttam Pal, Supriya Das, Khyati Bagga, Anupam Roy, Arpita Mrigwani, Nakul C. Maiti

**Affiliations:** Structural Biology and Bioinformatics Division, Council of Scientific and Industrial Research (CSIR)-Indian Institute of Chemical Biology (IICB), Kolkata, India; UMR-S665, INSERM, Université Paris Diderot, INTS, France

## Abstract

An amyloidogenic region (AR) in a protein sequence plays a significant role in protein aggregation and amyloid formation. We have investigated the sequence complexity of AR that is present in intrinsically disordered human proteins. More than 80% human proteins in the disordered protein databases (DisProt+IDEAL) contained one or more ARs. With decrease of protein disorder, AR content in the protein sequence was decreased. A probability density distribution analysis and discrete analysis of AR sequences showed that ∼8% residue in a protein sequence was in AR and the region was in average 8 residues long. The residues in the AR were high in sequence complexity and it seldom overlapped with low complexity regions (LCR), which was largely abundant in disorder proteins. The sequences in the AR showed mixed conformational adaptability towards α-helix, β-sheet/strand and coil conformations.

## Introduction

The available genome sequences and several computational methods have revealed a unique presence of some proteins which remain disordered under physiological condition and resemble their own functional states [1]–[9]. These proteins are known by different names like intrinsically disordered [10], natively denatured [11], natively unfolded protein and intrinsically unstructured proteins [3], [10]. The accepted convention is however intrinsically disordered protein (IDP). It comprises of 25–30% of eukaryotic proteome and ∼50% of eukaryotic proteins contain long disorder regions [12]. The IDPs lack any well-defined three dimensional folded structures in solution and structurally they remain as an ensemble of interconverting conformations under physiological conditions [13]–[15]. The lack of a rigid and folded stable structure may provide large plasticity to IDPs to interact efficiently with different targets, as compared to a globular protein with limited conformational flexibility [16], [17]. These characteristics possibly aid good efficacy to IDPs to be involved in different pathological and biochemical functions [5], [6], [13], [16], [18]–[20]. The functional domain varies from DNA binding to cell cycle regulation, membrane transport, different molecular recognition processes, and other important cellular functions [19], [21]–[23].

In addition to IDPs' important role in cellular activity, the inherited structural disorder plays an important role in the formation of protein assembly structure [24]. The structural disorder and flexibility of IDPs are also linked to formation of amyloid aggregates that is implicated in several human disorder such as Parkinson's disease, Alzheimer's disease, type II diabetes and others [25]–[30]. The major protein component of fibrillar deposits found in Parkinson's disease is a disordered protein, α-synuclein [25]–[30]. Alzheimer's disease is directly linked with production of ordered fibrillar structure of peptide Aβ42. Thus several neurological disorders are linked to formation of amyloid fibrils and their deposition in various cellular organs.

However, it is not very clear how normally soluble disordered proteins/peptides are converted into amyloid fibre that possesses compact β-sheet structure. It has been also further observed and presented in many in vitro experiments that some structured proteins convert to amyloid fibrils under solution conditions where the proteins attained partial disordered structure [31], [32]. Experimental study and many computational analyses showed that short sequence stretches in proteins may be responsible and act as nucleating centres for amyloid fibril formation [33]–[36]. These regions are often known as amyloidogenic regions (ARs). Amyloidogenic sequences of six to eight residues when inserted in the C-terminal hinge loop of RNase A, the enzyme shows amyloidogenicity and forms amyloid fibres [34]–[36]. Presence of such regions in many water soluble proteins has been suggested by Dobson [36], [37] and others [38]. According to ‘amyloid stretch hypothesis’ [35], a short amyloid stretch (equivalent to AR) in a certain solution condition triggers the aggregation process. Mutation or reshuffling in this regions leads to decrease or total absence of such aggregation [33], [39]. Thus AR often acts as a nucleation center and governs protein aggregation that eventually leads to formation of β sheet rich amyloid fiber.

The IDPs are also rich sequences with biased amino acid residues in a stretch, often known as low complexity regions (LCRs). These regions may also play a critical role in protein stability and energetic of fibril formation [1], [40]–[47]. LCRs are usually of two types: a majority of LCRs is composed of mixed polar and charged amino acid (aa) residues and the presence of such regions enhances protein solubility and mobility in solution. Second type of LCR is a repeat of one/two sequence which is prone to form amyloid fiber. A good example of such region is a stretch of Glu (polyGlu) [48]. Thus the presence of LCR modulates the solubility and amyloidogenicity of disordered proteins [45], [49], [50].

The composition, content and distribution of ARs and LCRs in a protein sequence, therefore, may have a certain role in protein aggregation and amyloidogenicity. However, no major investigation has been carried out regarding sequence complexity of ARs and their spacing among LCRs which are commonly found in IDP sequences. In the present investigation, we computationally detected and analyzed the sequence composition and complexity, distribution pattern and structural aspects of ARs and LCRs in proteins those are deposited in DisProt and IDEAL databases [4], [50], [51]. About 8% residue is found to be in AR and the average length of the region is 8 residues. Further we have found that the sequences in AR are highly complex and they rarely overlap with LCR.

Among many recently developed computational approaches and algorithms, we have used Waltz method that is developed by Maurer-Stroh et al. [52]–[56] to predict the ARs. The Waltz algorithm uses a position specific scoring matrix (PSSM) and combined physical properties and structural aspects of protein residues to identify AR [40], [41], [57], [58]. Computation tool SMART is used to predict the sequence complexity parameters. We have measured the structural propensity of the residues in AR by APSSP2 algorithm which is freely available in the World Wide Web [59], [60].

## Materials and Methods

### Selection of Intrinsically Disordered Proteins

DisProt database release 5.6 (http://www.disprot.org/) provides a set of proteins with different degree of disorderness [4]. It gives the name of the protein, accession codes, aa sequence, location of the disordered region(s), and methods used for structural (disorder) characterization. DisProt analysis also reveals biological function(s) of each disordered regions. Sequences of each protein were retrieved in FASTA format. Length, the aa composition, residue characteristics such as total number of positive and negative residues and theoretical isoelectric point (PI) were computed using the ProtParam tool of ExPASy Proteomic server (http://us.expasy.org/tools/protparam.html). The total charge of the proteins was calculated by ‘protein calculator’ server (http://www.scripps.edu/~cdputnam/protcalc.html).

Additional disordered proteins were selected from IDEAL data set that contained experimentally verified IDPs [51]. The structural disorder of the proteins was varied from 0 to 100%. The proteins with (−1)% disorder were excluded. Structural disorder was further calculated using IUPred algorithm, which is available at http://iupred.enzim.hu [61]. Protein disorderness was estimated by counting the number of residues in disordered regions in a protein as predicted by IUPred and it was divided by the length of the protein sequence followed by multiplication with 100.

### Calculating LCR and AR

Protein sequences obtained from DisProt and IDEAL were used to calculate both the LCR and AR. The content of LCR of an individual protein was predicted by SEG method as implemented in SMART (simple modular architecture research tool) [40], [62], a web based server available at http://www.bork.embl-heidelberg.de/Modules/sinput.shtml. Default SEG parameters were used for finding the LCR. The SEG method detects LCRs based on the measurement of information content present in the complexity state vector [40]. The ratio of total number of aa residues in all the LCRs of a protein to the protein sequence length was used to calculate the content of low-complexity region in a particular protein. Amyloidogenic region of the proteins was identified by a web based computational tool Waltz [56], http://waltz.switchlab.org. The % content of residues in AR in a protein was measured by taking a ratio of sequences in all the ARs and the sequence length of the protein.

### Prediction of Secondary Structure

APSSP2 was used for the secondary structure prediction of each protein from their aa sequence [59]. The algorithm uses a sequence of amino acids as a query input and predicts the corresponding secondary structure with certain confidence level. Percentages of residues those prefer to be in α-helix, β-strand and coiled conformation were calculated by taking a ratio of total residues in a particular conformation to the sequence length of the proteins. Structural preferences of the residues in ARs and LCRs were obtained by selecting the respective sequence regions in the predicted structure of the protein. Percentage of AR/LCR sequence with a preference for a particular conformation was measured against the total number of AR/LCR sequence in the protein.

### Statistical Analysis

All the statistical analysis was performed in Wolfram Mathematica 8. Mean, standard error of mean (SEM), standard deviation (SD) were calculated for AR/LCR length and content. Stable distribution function (Text S1) with index of stability α, skewness parameter β, location parameter μ, and scale parameter σ was fitted to the data to show distribution pattern of AR/LCR length and the AR/LCR content in a protein. Bivariate probability distribution such as smoothed kernel density distribution was used to show the distribution of AR/LCR content with the protein length. To find the correlation between the AR/LCR content and protein sequence length negative hyperbolic equations were fitted to the data.

## Results

### Content of AR and LCR in Different Classes of IDPs

The DisProt database analysis revealed 221 human proteins and 432 nonhuman (other than human) proteins with different degree of disorderness. Table 1, Tables S1 and S2 list some of these proteins with their physicochemical properties. Additional 186 unstructured human proteins and 25 nonhuman proteins were obtained from IDEAL database (Tables S3 and S4). Tables S1, S2, S3, and S4 show the protein name, database ID and the % of protein disorder measured by IUPred. The tables also show the content (%) of AR and LCR in a particular group of proteins. Last two columns in the tables display the number of ARs found within 15 residues from the C- and N- terminal of the protein sequence and these are marked as ‘C’ and ‘N’ column, respectively. The DisProt database provides the content of structural disorder, however, the disorderness of all the proteins present in IDEAL and DisProt databases was calculated using IUPred server. The proteins from both the databases were arranged in a descending order of disorderness. The content (%) of AR sequences decreased with increasing order of structural disorder. However, a less number of LCR sequence was present in proteins with high content of structural elements.

**Table 1:** Some of the intrinsically disordered human proteins from DisProt database.

Sl No.	DisProt ID	Protein	Localization/source	Function/role†	PI	Sequence length	aa# (−,+,0)
**1**	DP00004_C002	Antibacterial protein LL-37	Secreted	Antibacterial activity	10.61	37	5,11,16
**2**	DP00016	Cyclin-dependent kinase inhibitor 1	Cytoplasm, Nucleus	Role cyclin-dependent kinase activity	8.69	164	23, 26, 81
**3**	DP00017	Cyclin-dependent kinase inhibitor 1C		Negative regulator of cell proliferation	5.39	316	31, 27, 202
**4**	DP00028	Eukaryotic translation initiation factor 4E-binding protein 1	Cytosol	Regulates eIF4E activity	5.32	118	14,12, 56
**5**	DP00039	Non-histone chromosomal protein HMG-17	Cytoplasm, Nucleus	Binds to nucleosomal DNA	10.00	89	14, 26, 40
**6**	DP00040	High mobility group protein HMG-I/HMG-Y	Chromosome, Nucleus	Processing of mRNA transcripts	10.31	107	15, 27, 36
**7**	DP00069	Vesicle-associated membrane protein 2	Synaptic vesicles	Membrane transport	7.84	116	12,13, 66
**8**	DP00070	α-synuclein	Membrane-bound in dopaminergic neurons	Dopamine release and transport	4.67	140	24,15, 73
**9**	DP000126	Tau [Isoform Tau-F]	Axons	Microtubule assembly and stability	8.24	441	56, 58, 200
**10**	DP00174	Stathmin	Cytoplasm	Regulation of the microtubule (MT)	5.76	149	36, 32, 52
**11**	DP00199	β-casein	Secreted	Modulate surface properties of the casein micelles	5.52	226	20,15, 131
**12**	DP00214	Osteopontin	Secreted	cell-matrix interaction	4.37	314	75, 29, 103
**13**	DP00219	Protein phosphatase 1 regulatory subunit 11	Widely expressed	Inhibitor of protein phosphatase 1	6.52	126	20,19, 51
**14**	DP00287	Tumor suppressor [Isoform 1]	Cytoplasm	Involved in the ubiquitination	4.70	213	41, 23, 114
**15**	DP00332	Bone sialoprotein 2	Secreted	Cell attachment	4.12	317	76, 23, 103
**16**	DP00357	Thymosin β-4	Cytoplasm	Organization of the cytoskeleton	5.02	44	11, 9, 13
**17**	DP00372	Uncharacterized protein C8orf4		Apoptosis	10.14	106	13, 24, 42
**18**	DP00510	Nuclear protein 1	Nucleus	Proapoptotic stimuli	9.98	82	10, 15, 34
**19**	DP00521	Securin	Cytoplasm, Nucleus	Chromosome stability	6.18	202	27, 26, 105
**20**	DP00546	Huntingtin-interacting protein K [Isoform 1]			5.35	175	34, 29, 81
**21**	DP00555	β-synuclein	Cytoplasm	Regulator of SNCA aggregation process	4.41	134	28, 13, 68
**22**	DP00592	Purkinje cell protein 4	Cytoplasm, Nucleus	Nervous system development	6.21	62	11,11, 23
**23**	DP00617	26S proteasome complex subunit DSS1		Proteolysis	3.81	70	27, 5, 26
**24**	DP00630	γ-synuclin	Cytoplasm	Neurofilament network integrity	4.89	127	23, 17, 56
**25**	Aβ42	APP(Amyloid precursor protein)	Cytoplasm	Alzheimer disease	5.31	42	6, 3, 25

# : −, + and 0 represent number of negative (−), positive(+) and neutral amino acids in the protein sequence, respectively.

† : from UniProt database and reference therein.

Their localization, function, PI, sequence length and amino acid compositions are listed.

**Figure 1:**
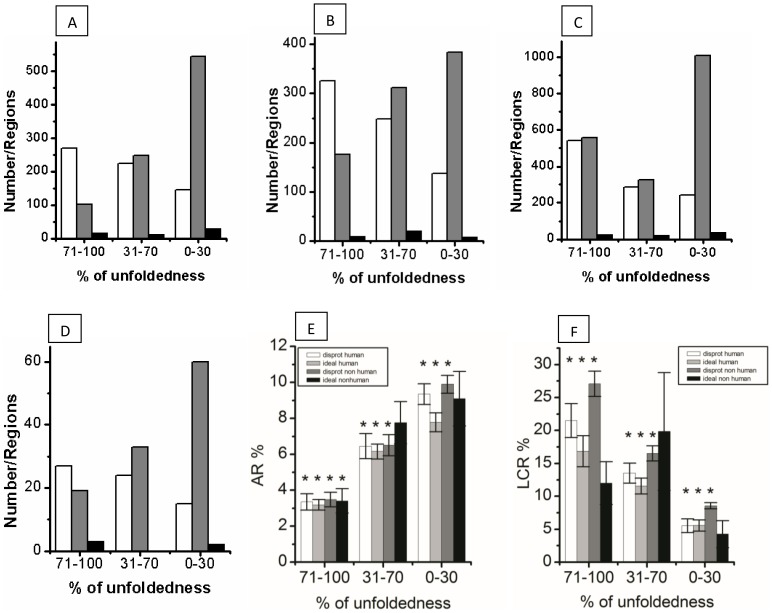
Content of AR and LCR sequences in different classes of disordered proteins. (A), DisProt human; (B), IDEAL human; (C), DisProt nonhuman and (D), IDEAL nonhuman. White bar signifying the LCR region, gray bar signifying the AR region and black bar signifying the overlapped region of AR and LCR. (E and F), Percentage of AR and percentage of LCR sequences in different group of disordered proteins, respectively. Bottom-axis in all the plots represents the three groups of disordered proteins with different degree of disorderness, PDP (0–30% disorder), MDP (31–70% disorder) and LDP (71–100% disorder). In (E) and (F), asterisks indicate the statistically significant difference from that of other groups (see Table S5).

Based on the calculated disorderness, the proteins in each type (human/nonhuman) of proteins were grouped into three categories as suggested in previous report [63]. Proteins with 71–100% structural disorder were grouped as largely disordered proteins (LDPs). Moderately disordered proteins (MDPs) possessed 31–70% sequences in disorder region(s) and the remaining proteins with less than 30% sequences the disorder segment were grouped as partially disordered proteins (PDPs). Sequence details of the AR and LCR in this group of proteins are shown in Table 2. Figure 1 displays the graphical view of the analysis. The number of LDPs was less compared to MDPs and PDPs. Percentage content of amyloidgenic proteins (proteins that contained at least one AR) was also found to be less in LDP group. To gain confidence about this analysis, a t-test was performed based on sequence content (%) in an individual protein of each group (LDP, MDP and PDP). Confidence level was gained from the respective p-values as given in Table S5.

**Table 2:** Content of AR and LCR sequences in different class of disordered proteins.

Database/Type	Class	Total number of proteins	Amyloidogenic proteins (count)	Amyloidogenic proteins (%)	AR (count)	AR (%)	LCR (count)	LCR %	Overlap regions (count)
**DisProt/Human**	LDP	56	39	69.64	102	3.35	269	21.49	15
	MDP	58	53	91.38	248	6.45	223	13.52	11
	PDP	107	99	92.52	544	9.35	146	5.51	27
	Total	221	191	84.51	894	7.22	638	13.49	53
**DisProt/Nonhuman**	LDP	124	70	56.45	556	3.47	542	27.08	22
	MDP	101	82	81.19	325	6.51	286	16.52	17
	PDP	207	188	90.82	1008	9.89	243	8.56	35
	Total	432	340	78.70	1889	7.26	1071	15.74	74
**IDEAL/Human**	LDP	45	39	86.67	176	3.18	325	16.86	9
	MDP	65	61	93.85	311	6.16	248	11.56	20
	PDP	76	75	98.68	384	7.78	137	5.56	7
	Total	186	175	93.07	871	6.10	710	10.39	36
**IDEAL/Nonhuman**	LDP	8	8	100.00	19	3.40	27	12.00	3
	MDP	7	7	100.00	33	7.75	24	19.84	0
	PDP	10	9	90.00	60	9.09	15	4.26	2
	Total	25	24	96.00	112	6.89	66	11.10	5

LDP, 71–100% disordered protein; MDP, 31–70% disordered protein; PDP, <30% disordered protein.

Table 2 and Tables S1, S2, S3, and S4 show that some of the proteins in each group contained no AR. For instance, among 221 human proteins in DisProt database, 191 (∼86%) proteins were amyloidogenic and each contained at least one AR. 30 human proteins contained no ARs. The number of amyloidogenic proteins was maximum (93%) for PDPs. However, the value decreased to 70% for the LDPs. A similar trend was observed with nonhuman proteins as presented in Table 2 and Table S2. Analysis of protein sequence from IDEAL database also revealed a similar trend in the content of amyloidogenic protein in different group of proteins (Table 2 and Table S3). Percentage of sequences in low complexity region (LCR) in each and individual protein in DisProt and IDEAL databases are also given in Tables S1, S2, S3, and S4. A group wise distribution of the LCRs is presented in Figure 1 and Table 2. The content of LCR sequence (%) was maximum in LDPs and a little more than 20% of the sequence was found in LCR regions in human proteins found in DisProt. The content of LCR sequences was found to increase with the decrease of structural disorder. Nonhuman DisProt proteins contained slightly higher percentage (16%) of LCR sequences than the proteins in human category. The LCR sequence content in proteins of IDEAL database was less than the DisProt proteins. The content of LCR was least in PDPs. P-values from the t-test of some of the above comparison are given in Table S5.

The sequence length of the AR/LCR and their content varied from protein to protein. Table 3 and Table S6 provide the sequence detail of the ARs, LCRs and the overlap regions between the two regions (AR/LCR). The table provides information regarding AR/LCR length and sequence position of the regions and the percentage of AR/LCR sequences in an individual protein. Individual AR lengths varied from 5 to 34 residues. The content of AR sequences was between 0 to 44% (Tables S1, S2, S3, and S4). For example, the shortest protein, 37 residues long antibacterial LL-37 (DP0004_C002) contained no AR, tau with 441 amino acids enriched with 1.3% AR residues. DP00069 with sequence length of 116 was very rich in AR sequences (14%).

**Table 3:** LCRs, ARs (*) and overlap regions (†) in some of the human disordered proteins from DisProt data.

DisProt ID	LCR/AR	Protein length	AR (%)	LCR (%)
**DP00016**	GPRRGRDELG GGRRPG (81–96)	164	0	10
**DP00017**	RLLLAPRPVA VAVAVSPPLE PAAES (101–125)	316	0	43
	PSVPVPAPAS TPPPVPVLAP APAPAPAPVA APVAAPVAVA VLAPAPAPAP APAPAPAPVA APAPAPAPAP APAPAPAPAP DAAP (137–220)			
	AAGTAAASAN GAA (251–263)			
	VPAPCPSPSA APGVGSV (291–307)			
**DP00039**	KRKAEGDAKG DKAKVKDE (2–19)	89	0	62
	AKPAPPKPEP KPKKAPAKKG EKVPKGKKGK ADAGKEG (29–65)			
**DP00040**	SESSSKSS (2–9)	107	0	66
	KRGRGRPRKQ PP (23–34)			
	PKRPRGRPKG SKNKG (54–68)			
	KTRKTTTTPG RKPRGRPKKL EKEEEEGISQ ESSEEE (71–106)			
**DP00069**	ATAATAPPAA PAGEGGPPAP PP (3–24)	116	14	33
	IILGVICAII LIIIIV (97–112)			
	VICAIILIII IVYFSS (101–116)*			
	VICAIILIII IV (101–112)†			
**DP00070**	KAKEGVVAAA EKTK (10–23)	140	4	21
	EGVLYV (35–40)*			
	VTNVGGAVVT GVTAVA (63–78)			
**DP00126**	SKSKDGTGSD DKKAKGADGK TKIAT (129–153)	441	1	17
	PAKTPPAPKT PPSSGEPPKS GDRSGYSSPG SPGTPGSRSR			
	TPSLPTPPTR EP (172–223)			
	KVQIIN (274–279)*			
**DP00174**	AFELI (19–23)*	149	3	0
**DP00199**	VLILACLVAL A (3–15)	226	0	38
	ETIESLSSSE ESITE (17–31)			
	HEDQQQGEDE HQD (41–53)			
	LPLAQPAVVL PVPQP (82–96)			
	LHLPLPLLQP LMQQVPQPIP Q (139–159)			
	LLLNQELLLN (196–205)			
**DP00214**	SHDHMDDMDD EDDDDHVDSQ DSIDSNDSDD VDDTDDSHQS	314	0	20
	DESHHSDESD E (81–131)			
	EFHSHEFHSH E (272–282)			
**DP00219**	ETVTETTVTV TTE (10–22)	126	0	37
	ESSTESDEEE EE (72–83)			
	PTPTTPPQPP DPSQPPPGPM Q (105–125)			
**DP00287**	EAEVGAEEAG VEEYGPEEDG GEESGAEESG PEESGPEELG	213	8	23
	AEEEMEAG (10–57)			
	SQVIF (72–76)*			
	IFANITLPVY TL (147–158)*			
**DP00332**	GSSDSSEENG DDSSEEEEEE EETSNEGEN NEESNEDEDS EAENTT (62–106)	317	3	41
	KEKESDEEEE EEEEGNENEE SEAEVDENE (145–173)			
	TGANAEGTTE TGGQGKGTSK TTTSPNGG (207–234)			
	GKTTTVEYEG EYEYTG (252–267)			
	GQGYDGYDGQ NYY (302–314)			
	GQNYYHHQ (310–317)*			
	GQNYY (310–314)†			
**DP00372**	HQAIIM (7–12)*	106	17	0
	AVGNIF (35–40)*			
	IIFAID (66–71)*			
**DP00510**	EDEDSSLDES DLYSL (18–32)	82	0	31
	GGGGRKGRTK RE (38–48)			
**DP00521**	ATLIYV (2–7)*	202	3	5
	PPSPVKMPSP P (163–173)			
**DP00546**	GAERRCGPGP APPPPRAEA (16–34)	175	5	21
	RRSREQKAKQ EREKELAK (116–133)			
	VEAL IALTN (167–175)*			
**DP00555**	EGVLYV (35–40)*	134	8	28
	GAGNIA (73–78)*			
	EEVAQEAAEE PLIEPLMEPE GESYEDPPQE EYQEYEPE (96–133)			
**DP00592**	AAVAIQ (42–47)*	62	10	0
**DP00617**	LLEEDDEFEE F (12–22)	70	0	36
	VWEDNWDDDN VEDD (38–51)			
**DP00630**	AVSEAVVSSV NTVATKTV (65–82)	127	0	30
	QQEGEASKEK EEVAEEAQSG (106–125)			
**Aβ42**	KLVFFA (16–21)*	42	29	0
	GGVVIA (37–42)*			

Sequence positions are given in the parentheses. Single letter code is used to represent individual aa residues.

In contrast to ARs, most of the LCRs were 8–40 residues long. The shortest LCR was 8 residues long. One such region was detected in DP00040. The largest LCR of 84 residues long was detected in DP00017. LCRs in tau (DP00126), for instance, occupied 17% of its total sequences. More than 35% residues in β-casein (DP00199) and regulatory subunit 1 (DP00219) were in LCRs.

### Statistical Analysis

Statistical analysis was carried out to reveal the average of AR/LCR content (%) and the length of the two regions (AR/LCR) in human proteins. To obtain the statistical parameters, AR/LCR content in all the human proteins from DisProt and IDEAL databases (Tables S1 and S2) was combined. The total number of proteins examined was 407 and the combined number of AR and LCR were 1765 and 1348, respectively, (Table 2).

**Figure 2:**
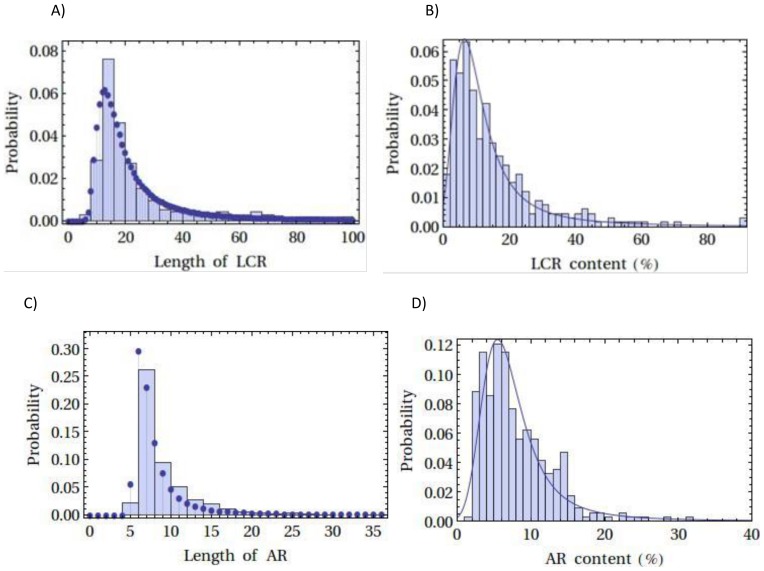
Probability distribution of LCR and AR lengths and percentages. Distribution of LCR lengths (A) and percentage of LCR (B) in LCR containing disordered proteins. C and D, respectively; represent probability distribution of AR lengths and AR content (%) of IDPs. Fitted statistical parameters are given in Table 4. Histograms of data are shown with a suitable bin size.

**Figure 3:**
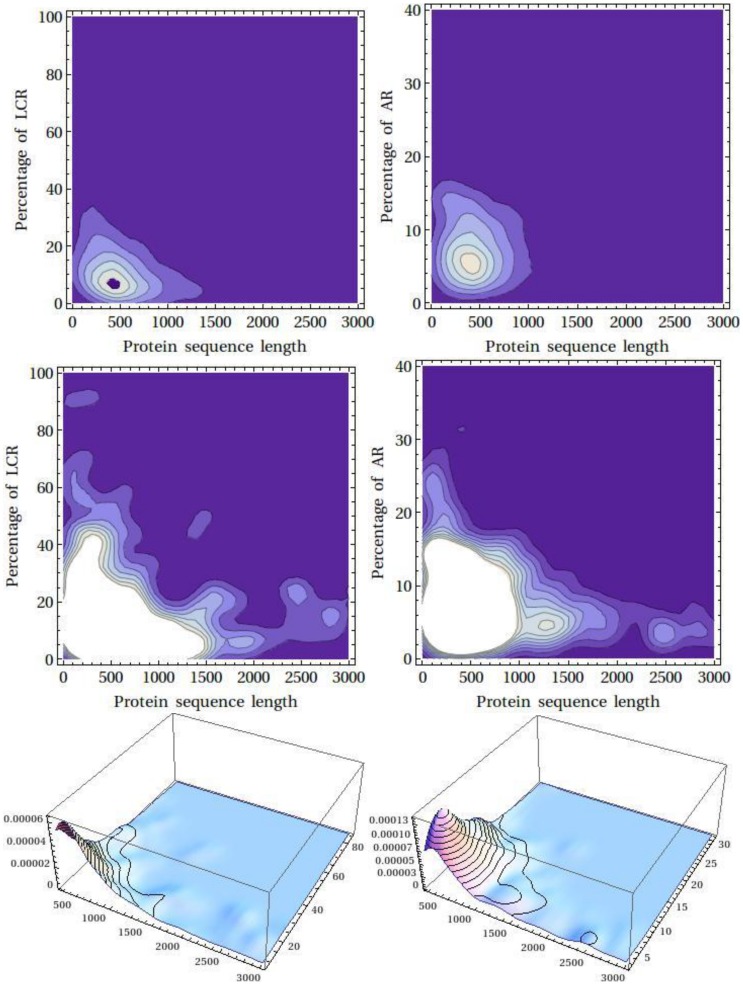
Smoothed kernel density estimation for the LCR and AR content in a protein. Left and right panel, respectively, represents the density for LCR and AR. The plots have been shown in two different clipping planes. Bottom figures show the smoothed 3D histogram for the AR and LCR.

A stable distribution function (see Materials and Methods and Text S1) was applied to the experimental data (detected ARs and LCRs). Figure 2 shows the frequency histogram and the fitted distribution function for both the LCR and AR. Table 4 reports the statistical parameter values estimated from the fit to ARs/LCRs. It was found that the statistical population (% of AR/LCR sequences) was characterized by a positive (and much larger than zero) value of the skewness coefficient. The mean value was ∼8% of sequences for the AR. A similar distribution fit was made to the available lengths of the ARs/LCRs as shown in Figure 3 and the mean value was about 8 residues for the AR and 34 residues for the LCR.

**Table 4:** Statistical analysis on AR/LCR length/content.

Stable distribution parameters	AR length distribution	AR percentage distribution	LCR length distribution	LCR percentage distribution
**Index of stability, α**	1.02	1.34	0.92	1.08
**Skewness parameter, β**	0.99	0.99	0.99	0.99
**Location parameter, μ**	6.55	9.73	14.99	9.73
**Scale parameter, σ**	0.94	2.24′	4.67	2.24

Stable distribution function fitting parameters.

**Figure 4:**
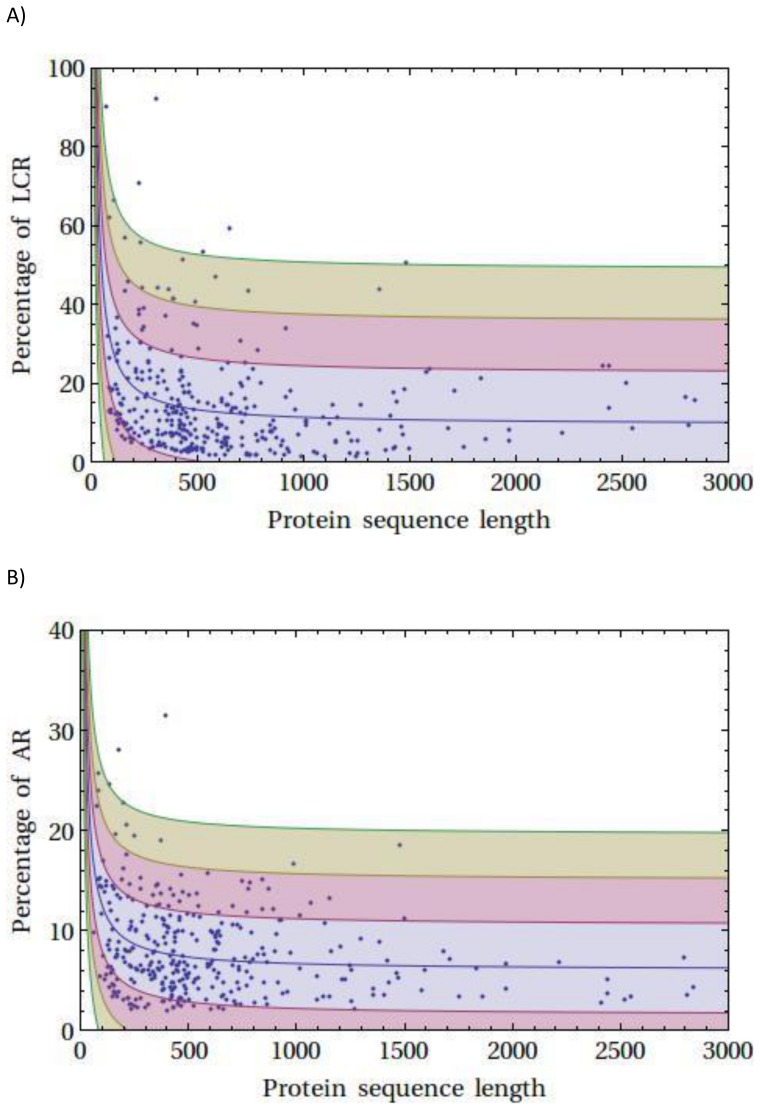
Correlations between content of LCR and AR sequence with the protein length. (A) Correlations between content of LCR sequence with the protein length. No significant correlation could be obtained for the LCR content in a protein sequence. The figure shows a negative hyperbolic fit (y = 9.44056+1926.61/x; R^2^, 0.113058) with standard deviation bands (at 1σ, 2σ, and 3σ). (B) Correlations between content of AR sequence with the protein length. No significant correlation could be obtained for the AR content in a protein sequence. The figure shows a negative hyperbolic fit (y = 6.05937+651.62/x; R2, 0.112173) with standard deviation bands (at 1σ, 2σ, and 3σ).

Figure 3 shows the smoothed kernel density estimation for the LCR/AR content in a protein (left and right panel, respectively). The plots have been shown in two different clipping planes. Bottom figure shows the smoothed 3D histogram. The smoothed kernel density estimation plot shows a distinct peak suggesting ∼8% AR content in a ∼400 aa long protein and indicated that the detected proteins in the two databases populated at ∼400 aa long and largely contributed to the estimate of average content of the AR and LCR. No correlation could be observed between the AR/LCR content and protein length (Figure 4). Although at deeper clipping plane it suggested a negative hyperbolic fit i.e. with the increase in protein length there is decrease in the AR/LCR content. However, no significant fit could be obtained to validate this assumption.

### Sequence Aspects of AR and LCR

One interesting observation was that a major number of proteins contained both the AR and LCR, however, the two regions rarely overlapped with each other (Figure 1, Tables S1, S2, S3, and S4, Table 3 and Table 5). For instance, DisProt human proteins contained 894 ARs and 638 LCRs, however, only 53 occurrences of sequence overlapping between the two regions were observed and in most of the cases the overlap was partial (Table 5). A LCR with residues 97–112 in DP00069 overlapped with C-terminal AR of residues 101–116, and the overlapping region contain 12 residues. Whereas in DP00332, LCR with residues from 302–314 overlapped with an AR (310–317). Only four residues were found in the overlapping region. Similarly four ARs from DP00119, DP00551, DP00643_A002 and DP00683 partially overlapped with the LCRs. In other group of proteins also a similar result was obtained. Among 1889 AR regions in DisProt nonhuman proteins, only 74 ARs overlapped with the LCRs. In an average, ∼3% of the AR sequences overlapped with the LCR sequences. These observations clearly indicated that the residues in AR were very complex and rarely overlapped with the LCR.

**Table 5:** Overlapping regions in DisProt human proteins.

Disprot ID		LCR/AR overlap region
**DP00069**	LCR	IILGVICAIILIIIIV---- (97–112)
	AR	----VICAIILIIIIVYFSS (101–116)
**DP00332**	LCR	GQGYDGYDGQNYY--- (302–314)
	AR	--------GQNYYHHQ (310–317)
**DP00119**	LCR	LLILLSVALLALSSAESSSEDVSQEESL---- (2–29)
	AR	--------------------------SLFLIS (28–33)
**DP00551**	LCR	ALLLLLFLHLAFL (10–22)
	AR	--LLLLFLHLAFL (12–22)
**DP00643_A002**	LCR	VILRLLRYIVRLVWR-- (122–136)
	AR	----LLRYIVRLVWRMH (126–138)
**DP00683**	LCR	LVSVYNSYPYYPYLY- (210–224)
	AR	LVSVYNSYPYYPYLYC (210–225)
**DP00012**	LCR	-----FNSSAFFFSGFFVVFLSV----------- (305–322)
	AR	AYVRYFNSSAFFFSGFFVVFLSVLPYALIKGIIL (300–333)
	LCR	IQLLLIVIGAIAVVAVLQ (995–1012)
	AR	-QLLLIVIGAIA------ (996–1006)
	LCR	-IFVIFFIAVTFISI-- (1106–1119)
	AR	MIFVIFFIAVTFISILT (1105–1121)
**DP00074**	LCR	AAYEFNAAAAANA (58–70)
	AR	AAYEFN------- (58–63)
	LCR	LTLQQQHQRLAQLLLIL- (495–511)
	AR	-----------QLLLILS (506–512)
**DP00099**	LCR	---TIITPPTPIIP (336–346)
	AR	AGWTIIT------- (333–339)
**DP00162**	LCR	TTGVVTVIVILIAIAALGALILG----- (9–31)
	AR	-------IVILIAIAALGALILGCWCYL (16–36)
**DP00191**	LCR	LLLLLFL-- (8–14)
	AR	-LLLLFLKS (9–16)
**DP00231**	LCR	------QTPQGQQGLLQAQNLLTQLPQQ (210–231)
	AR	AQFIISQ--------------------- (204–210)
**DP00272**	LCR	--------LALADALATSTL (112–123)
	AR	ATNIYIFNLA---------- (104–113)
**DP00282**	LCR	KNNWNIEDNNIKN (1132–1144)
	AR	-NNWNIE------ (1133–1138)
**DP00306**	LCR	----ITILIIALIAL------ (51–61)
	AR	NVVFITILIIALIALSVGQYN (47–67)
**DP00307**	LCR	LEQILEYELLLIQQL------ (140–154)
	AR	-------ELLLIQQLNFHLIV (147–160)
**DP00311**	LCR	AVAGLVLVALLAILV---- (232–246)
	AR	--------ALLAILVENWH (240–250)
**DP00314**	LCR	PKLPDDTTFPLPPPRPK----- (149–165)
	AR	----------------KNVIFE (165–170)
**DP00317**	LCR	TEKRKKRSTKKE---------- (301–312)
	AR	-----------EVFNILQAAYV (312–322)
**DP00324**	LCR	GGNFGGRSSGPYGGGG--- (329–344)
	AR	--------------GGQYF (343–347)
**DP00338**	LCR	MILFLIMLVLVLF--- (20–32)
	AR	-ILFLIMLVLVLFGYG (21–35)
**DP00339**	LCR	MILFLIMLVLVLF--- (20–32)
	AR	-ILFLIMLVLVLFGYG (21–35)
	LCR	GDFYYLGGFFGG (261–272)
	AR	GDFYYLGGFFG- (261–271)
**DP00356**	LCR	NNQYFNHHPYPHNHYMP (120–136)
	AR	NNQYFN----------- (120–125)
**DP00381**	LCR	-----NNTQTTTHLQPLHHP (819–833)
	AR	ELNNINNTQ----------- (814–822)
**DP00406**	LCR	LQALYALQALVVTL- (1522–1535)
	AR	LQALYALQALVVTLE (1522–1536)
**DP00428**	LCR	-------LELCRRRSLLEL (130–141)
	AR	NDFVFVVLEL--------- (123–132)
**DP00448**	LCR	LVVKTALKLLLVFV--- (217–230)
	AR	--------LLLVFVEYS (225–233)
**DP00464**	LCR	KKLKEKKDELD--------- (45–55)
	AR	---------LDSLITAITTN (54–64)
**DP00466**	LCR	SPPVILLISFLIFLIV- (237–252)
	AR	---VILLISFLIFLIVG (240–253)
**DP00467**	LCR	AKPNATTANGNTALAIA (785–801)
	AR	-----------TALAIA (796–801)
**DP00503**	LCR	---------LLIILFIIVPIFLLL (167–181)
	AR	KDGIIMIQTLLIILFIIVPIFLL- (158–180)
**DP00508**	LCR	LAVLILAIILL------ (7–17)
	AR	LAVLILAIILLQGTLAQ (7–23)
**DP00519**	LCR	-----SSGAKSPSKSGA (1355–1366)
	AR	KAVEFSS---------- (1350–1356)
	LCR	LEELEKERSLLLADLDKEEKEKD----------- (134–156)
	AR	---------------------KDWYYAQLQNLTK (155–167)
**DP00520**	LCR	KSPKGSGKPPGVPASSKSGK------ (332–351)
	AR	-------------------KAFSYYL (351–357)
**DP00553**	LCR	ASLLFLNVLAFAAL- (716–729)
	AR	ASLLFLNVLAFAALY (716–730)
**DP00574**	LCR	GPGRLEREAAAAAATTPAPTAGAL--- (52–75)
	AR	--------------------AGALYSG (72–78)
	LCR	-----SGSEGDSESGEEEELGAE (77–94)
	AR	AGALYSG---------------- (72–78)
**DP00616**	LCR	LVFLVLLFLGALGLCLA (3–19)
	AR	---LVLLFLGA------ (6–13)
**DP00628**	LCR	LRELSELSLLSL-- (235–246)
	AR	--------LLSLYG (243–248)
**DP00632**	LCR	YSTYSQAAAQQGYSAYTAQ (6–24)
	AR	-----------GYSAYTA- (17–23)
	LCR	---SYTQAQTTATYGQTAYATSYGQPPTGYTTPTAPQA (51–85)
	AR	TDVSYTQAQTTATYGQTAYATSYG-------------- (48–71)
	LCR	QPVTAPPSYPPTSYSSTQPTSYDQSSYSQQNTYG…QSS (182–266)
	AR	----------------------------QQNTYG------ (210–215)
**DP00633**	LCR	-LQAYQQRLLQQQ (2257–2268)
	AR	SLQAYQ------- (2256–2261)
**DP00641**	LCR	AALLWLLLIAAA-- (5–16)
	AR	AALLWLLLIAAAFS (5–18)
**DP00666**	LCR	IILLLLVLLIL-- (1130–1140)
	AR	-----LVLLILCF (1135–1142)
**DP00670**	LCR	AVAAAAIFVIIIF- (314–326)
	AR	--AAAAIFVIIIFY (316–327)
**DP00706**	LCR	GKGDSSGFSSYSGSSSSGSSISSARSSGGGSSG…AGS (58–105)
	AR	------GFSSYS--------------------------- (64–69)
	LCR	GYSQVSYSSGSGSSLQGASGSSQLGSSSSHSGNSGS…GSA (111–175)
	AR	--SQVSYSS--------------------------------- (113–119)

Length and sequence positions are given in the parentheses. Single letter codes are used to represent individual aa residues. Overlapping regions are aligned. Only the proteins with AR/LCR overlapping regions are shown.

**Figure 5:**
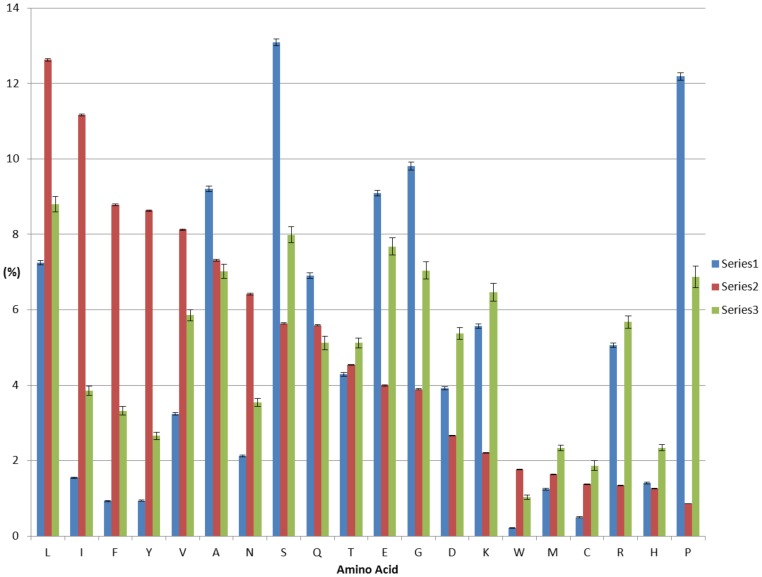
Content of different types of aa residues present in the LCR, AR and total proteins. The panel compares the percentage of individual aa residues in the LCR (Series 1, blue), AR (Series 2, red), and total protein (Series 3, green). X-axis started with the most abundant residues in the AR. The amino acid residues are presented with a single letter code along the bottom axis.

We also calculated average content of different types of amino acid residues in both the AR and LCR. Figure 5 displays the average content of different types of residues present in the AR, LCR and total proteins. A major fraction of the AR residues was hydrophobic and Leu was the most abundant (12.6%) residue. Other major residues in the region were Ile (11.2%), Phe (8.8%), Tyr (8.6%), Val (8.1%), Ala (7.3%). The AR regions were depleted in Pro, Lys, His and others. A major number of residues in the LCR was hydrophilic in nature and the regions were enriched with Ser (13.1%), Pro (12.1%), Gly (9.8%) and Ala (9.2%).

**Figure 6:**
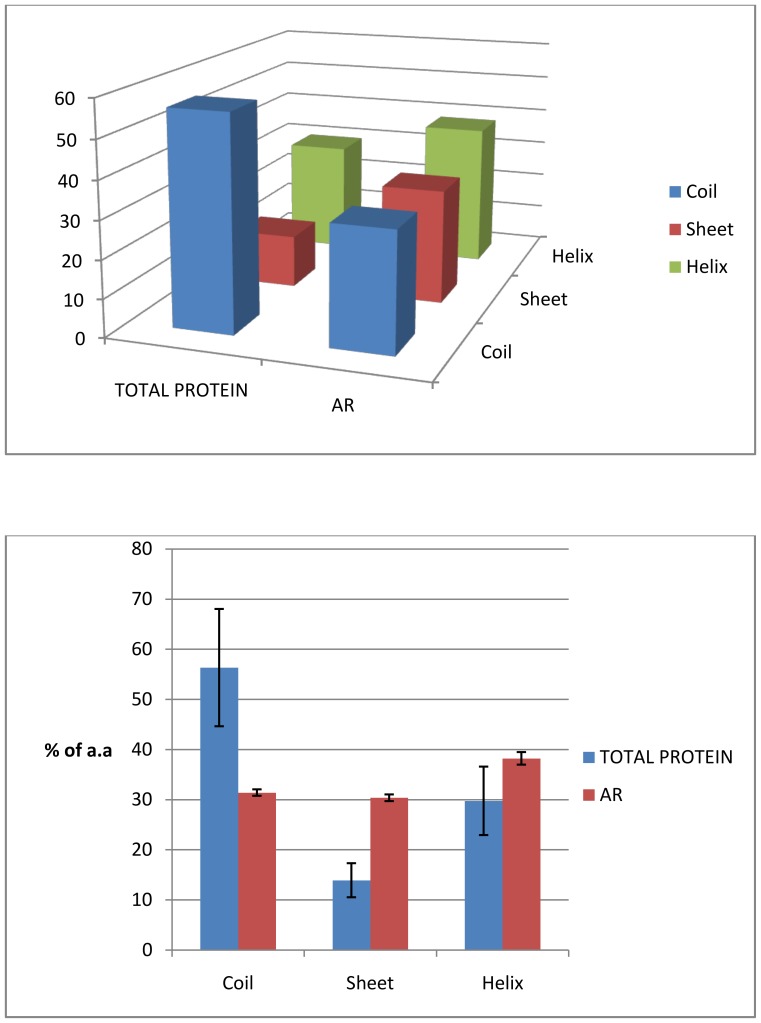
Comparison of the conformational preferences of residues in the ARs with that of total protein. A 3D plot shows the percentage of residues with conformational preference for α-helix (green), β- strand/sheet (red) and coil (blue) for total proteins and their ARs as represented in X-axis. Lower panel shows the 2D plot of the above data along with the error limits.

The structural propensities of residues in the ARs were measured using the APSSP2 algorithm (see Materials and Methods). The analysis showed that the conformational preference of the AR residues was not confined to any particular structure, rather in average a mixed structural preference of the AR residues was observed in all three groups of proteins. Figure 6 displays the overall structural heterogeneity of the AR sequences present in human (DisProt) proteins. The average number of sequence that preferred α-helical conformation was ∼38%. Preferences for β-sheet/strand and coil conformations were ∼31% and ∼32%, respectively. This result indicated that all of the sequences in the ARs did not favour β-conformation. When compared with total protein sequence present in the same group of proteins, about 56% residues preferred coil conformation and ∼30% residues showed structural propensity towards α-helical conformation. Remaining 14% favoured β-sheet/strand conformations. Number of residues that preferred β-sheet component increased substantially in the ARs, however, large fraction of the AR residues (38%) favoured α-helical conformation.

## Discussion

It is known from previous investigations that AR acts as a key for several protein aggregations and amyloid fibril formation. In this report we detected ARs by using Waltz algorithm and analyzed computationally the sequence complexity, conformational preference and the distribution of ARs in disordered human proteins present in Disprot and IDEAL databases. There are several methods to detect ARs [56], [64]–[66]. Some important algorithms and software to predict aggregation aspects of proteins are Tango [55], Waltz [56], PASTA [67]–[70], Aggrescan [71], SALSA [72], Zyggregator [73], AmylPred [64], FoldAmyloid [74]. The ability of the protein sequences to form β-strands/sheets is a predominant feature in most of these algorithms. PASTA was developed based on hidden β-propensity of the protein sequences [67]–[70]. Aggrescan software was based on an aggregation propensity scale for the 20 natural amino acids [71]. This method stressed that short and specific sequence stretches were responsible for protein aggregation. Based on average packing density of the aa residues, FoldAmyloid identified a sequence pattern that could promote amyloid fibril formation [34]. Waltz methodology was used in this investigation because many of its selected regions were experimentally verified and the method was better capable to differentiate amyloid fiber formation and amorphous aggregates [56].

The investigation revealed that more than ∼80% disordered human proteins (DisProt and IDEAL databases) possessed at least one AR, indicating that a significant number of disordered proteins were amyloidogenic. Waltz detected ARs from a large number of proteins in DisProt and IDEAL databases. The large number of data set helped to derive, along with discrete analysis (Table 6), statistical average of AR and LCR sequence percentage and the average of AR and LCR sequence length. Discrete analysis result of all groups of proteins is given in Table 2 and Table 6. The average values did not differ much with statistical analysis result (Table 4). However, the statistical values may be more acceptable to represent the average properties and composition of the LCRs and ARs.

**Table 6:** Discrete analysis.

Protein type	AR (%)			LCR (%)		
	Range	Mean	Median	Range	Mean	Median
**DisProt human**	0.43–31.50	8.36	6.98	1.41–91.94	15.86	10.21
**DisProt nonhuman**	1.20–44.00	9.27	7.50	1.30–96.80	16.80	12.20
**IDEAL human**	0.69–22.37	6.56	5.93	1.09–70.80	13.74	10.93
**IDEAL nonhuman**	1.08–17.53	7.03	6.69	1.67–70.67	13.15	8.14

Range, Mean, Median and Mode of AR and LCR sequence percentage in different group of proteins.

Percentage of amyloidogenic proteins was higher in the PDP groups. Thus the content of AR sequences was more in proteins with less structural disorder or in structured proteins. A similar observation was also made by Linding et al. [75]. These proteins contained less number of LCRs which were composed of less number of hydrophobic amino acids. LCR thus may have a significant role in protein aggregation process and amyloid formation. AR may be exposed to start the aggregation process and LCR regions could have certain role in the process. However, a large number of LCR along with a high content of polar amino acids and attenuated hydrophobicity may not allow the protein to misfold/fold further to gain β-sheet rich amyloid aggregate, in largely disordered proteins [3]. Therefore, the content of AR and LCR and the unique balance between the two regions are very crucial for protein stability (for disordered proteins) and amyloid formation. A proper solution condition may be needed based on the content of AR/LCR to unfold the region of structured proteins partially or fully to trigger amyloid fiber formation [76]. Nature may have designed the disordered proteins with a unique balance of AR and LCR sequences to provide stability and the ability to perform multifunction. However, an external disturbance or change in internal cellular condition may break this unique balance and could enhance protein aggregation and amyloid formation.

Most of the detected ARs in amyloidogenic proteins were six to eight residues long. We detected six residues long (residues 35–40) AR in α-synuclein. It was significantly shorter than the aggregation prone segment obtained by Der-Sarkissian et al. Zhang et al. showed four additional segments that might be involved in α-synuclein aggregation [72]. However, the used methods did not define adequately the characteristics of nucleation site of amyloid formation. Waltz allowed identification and better distinction between amyloid sequences from the protein segments that promote β-sheet rich amorphous aggregates, and that could be a possible reason of less number of AR regions found in this investigation.

Statistical analysis results and discreet analysis (Tables S1, S2, S3, and S4, Table 6) established that the content of AR sequences was not always proportional to the protein sequence length. It showed a negative hyperbolic correlation among the protein sequence length and the percentage of AR/LCR sequence (Figure 4). The reason of this was not known. Chiti et al. observed less aggregation propensity of proteins those were longer with respect to short proteins [77]. The longer proteins thus may have evolved with attenuation (low content) of ARs to reduce unwanted aggregation and fibril formation. It would be interesting, however, to test whether increasing number of ARs could enhance the aggregation kinetics or the quality of fibril formation in longer proteins.

In this regard, it was also important to know the conformational preferences of AR residues. We observed that aa residues in the ARs showed propensity towards α-helix, β-sheet/strand and coil conformations and all the residues were not very hydrophobic. Waltz, used in this investigation, did not fully rely on β-sheet structural propensity of the residues but was built on PSSM and on consideration of other physicochemical properties of the protein sequences. It allows some tolerance towards charged and polar residues with different hidden structural propensity. Proteins with diverse structural domains (β-sheet, α-helix, or random coil) including globular proteins were found to produce aggregates with fibrillar structure under certain solution condition [23], however, a crucial structural rearrangement often occurred during conversion of these proteins into amyloid fiber [78]. Thus slightly polar amino acids or the presence of LCR may play important role in structural reorganization.

Aggregation propensity and overall protein aggregation may also depend on the location of AR in the protein sequence, and how the ARs are surrounded by local excess of polar/charged amino acids or LCRs. Kar et al. recently showed that addition of a polyproline sequence to C-terminal side of polyGlu slowed aggregation of the peptide [48]. However insertion of the same residues to the N-terminal side of polyGlu caused very little effect on overall aggregation of the peptide. N-terminal residues in Huntingtin protein situated adjacent to the polyGlu sequence dramatically altered aggregation property of the peptide. However, position dependent role of LCRs, rich in polar and charged residues, on aggregation propelled by ARs was not known with certainty. According to amyloid stretch hypothesis the AR containing proteins were needed to be locally/partially unfolded to initiate and promote the process of amyloid fiber formation [35]. Thus the presence of LCR in a protein with less disorder may significantly alter the amyloid formation kinetics.

The IDPs play a vital role in molecular recognition process and the interaction has found to lead formation of structured protein complexes. A model of molecular recognition features or elements (MoRFs) has been proposed to define this interaction and the reorganization processes [79]–[82]. The MoRF model recognizes, in a disordered protein sequence, a linear region that undergoes a disorder-to-order transition upon binding to its partner. These regions are often referred as MoRFs. The regions could attain α-helices, form β-strands (β-MoRFs), irregular structures (ι-MoRFs), and a combination of all these structural elements upon binding to its partner. However, our analysis largely directed to find the amyloid forming region and the region of protein sequences that are sequentially less complex. Both the AR and LCR could be part of MoRFs and may be involved in molecular reorganization process. However, further analysis may be needed to address this issue.

One of the significant observations was that the AR sequences were highly complex. Our analysis with IDPs showed that ∼20% sequence was in the LCR and the value was close to the overall predicted value for SWISS-PROT database [41]. However most (greater than 97%, Table 2) of the AR sequences were not within the LCRs. It indicated complexity pattern of the AR sequences and confirmed the presence of less number of biased aa residues in the ARs. Some LCRs with one or more aa residues form stretches of a single amino acid, produce homopolymeric structure [41], [49], [40], [83] and became amyloidogenic [84]. However, we could detect in IDPs no such LCR which were polymeric in nature and amyloidogenic. Many prion proteins, e.g mammalian PrP, the yeast prions, Ure2p and Sup35 contain disordered stretches that also form beta sheet rich aggregates. These aggregate prone domains are also found to contain segments with low sequence complexity and often are enriched with Glu/Asp [85]–[88]. Thus prion proteins also contained both the ARs and LCRs. A test was performed with prion protein (P04156) and Huntingtin (P42858), however waltz methods could detect the palindromic region (residue 112–119) in P04156 and polyQ region in Huntingtin (P42858) only when ‘custom’ is used as the threshold in the analysis [56]. In our analysis, ‘best overall performance’ was used as the threshold and it missed the detection of above two amyloidogenic regions. We also analysed the content of ARs and LCRs in a group of proteins which were amyloidogenic and the amyloidogencity of the proteins were experimentally proven [56]. The list of the proteins and the analysis results are shown in Table 7. It includes protein like insulin, prion protein (P04156) and yeast protein Sup 35 (P05453). The observation was that the sequence overlapping of the AR and LCR were also very less (Table 7). This indicated that the ARs are compositionally highly complex. As such the sequence complexity and structural heterogeneity of the AR sequences was a vital observation. Also a few % of residues that overlapped with the LCR showed mixed structural propensity. The C terminal LCR in DP00069 that overlapped with the AR contained seven Ile (not at a stretch) and these residues showed preference for α-helical conformation. The overlapping sequences of AR and LCR, however, in DP00332 showed propensity towards random coil structure. Being a part of an AR both the overlapping regions was expected to induce aggregation in a certain solution condition. However, the LCR component may modulate the aggregation process in different way and the content may be changed depending on the solution condition [89]. Future experiments, starting with these overlapping ARs and LCRs, would enhance our understanding about how the sequence region composed of AR with low complexity sequences would modulate the protein aggregation process that lead to eventual formation of amyloid fiber.

**Table 7:** Content of ARs and LCRs in a group of known amyloidogenic proteins.

Name	UniProt ID	Sequence length	LCR	LCR (%)	AR	AR (%)	Overlapping sequences
**Insulin**	P01308	110	2–24	20.91	36–42	17.30	
					99–110		
**Apolipoprotein A1**	P02647	267			8–15	3.00	
**Cold shock protein cspB**	P32081	67			14–20	8.20	
					26–34		
					47–52		
**Acylphosphatase2**	P14621	99					
**Immunoglobulin G-binding protein G**	P06654	448	69–114	24.55			
			241–253				
			379–413				
			427–442				
**Alpha- synuclein**	P37840-1	140	10–23		35–40		
			63–78				
**PI3-kinase alpha**	P27986	724	79–102	7.18	72–78	6.40	
			303–314		263–269		
			533–548		290–296		
					331–336		
					401–406		
					483–495		
							
**Microtubule-associated protein Tau**	P10636	441			274–279	1.36	
**Cystatin-C**	P01034	146	2–33	21.92	10–20	22.60	10–20
					56–61		
					84–92		
					124–130		
**Ig kappa chain V-I region Rei**	P01607	108			32–37	20.40	
					45–53		
					71–77		
**Lysozyme C**	P00698	147			52–62	11.60	
					142–147		
**Major prion protein PrP**	P04156	253	50–94	38.74	8–17	19.40	240–252
			113–135		171–176		
			188–201		178–185		
			237–252		222–227		
					231–235		
					240–253		
**Sup35**	P05453	685	5–64	27.88	9–18	20.00	9–18
			68–113		31–36		31–36
			130–142		45–56		45–56
			164–209		69–74		69–74
			241–253		102–108		102–108
			398–410		260–266		
					278–285		
					304–313		
					426–445		
					471–476		
					527–538		
					566–571		
					584–596		

Proteins were selected from the reference 56.

## Conclusion

The current investigation was focused on sequence complexity and content of AR present in proteins which were partially or fully disordered. The study observed a very high sequence complexity of the ARs and the regions not commonly overlapped with the LCRs which were abundant in the protein sequence. The future investigation may examine experimentally whether a unique balance between the content of AR and LCR could provide a suitable stability to a monomeric disordered protein to remain in a solution state. It would be interesting to examine how the spacing of LCR and AR and, swapping of AR positions influence the energetic of amyloid fiber formation. It will enhance our understanding why some proteins favor aggregation in a certain environment and may add more information about the mechanism of amyloid formation which is linked to several pathological human disorders.

## Supporting Information

Text S1 **Stable distribution function.** Details of the statistical distribution function applied to AR/LCR length/content distribution.
(DOCX)

Table S1 **DisProt human proteins.** Protein name, database IDs and AR/LCR content measured by IUPred are listed. Last two columns in the tables display the number of ARs found within 15 residues from the C- and N- terminal of the protein sequence and these are marked as ‘C’ and ‘N’ column, respectively.
(XLSX)

Table S2 **DisProt nonhuman proteins.** Protein name, database IDs and AR/LCR content measured by IUPred are listed. Last two columns in the tables display the number of ARs found within 15 residues from the C- and N- terminal of the protein sequence and these are marked as ‘C’ and ‘N’ column, respectively.
(XLSX)

Table S3 **IDEAL human proteins.** Protein name, database IDs and AR/LCR content measured by IUPred are listed. Last two columns in the tables display the number of ARs found within 15 residues from the C- and N- terminal of the protein sequence and these are marked as ‘C’ and ‘N’ column, respectively.
(XLSX)

Table S4 **IDEAL nonhuman proteins.** Protein name, database IDs and AR/LCR content measured by IUPred are listed. Last two columns in the tables display the number of ARs found within 15 residues from the C- and N- terminal of the protein sequence and these are marked as ‘C’ and ‘N’ column, respectively.
(XLSX)

Table S5 **The t-test results for the comparison of different group of proteins.** The p-values were obtained using inbuilt program in Microsoft Office Excel.
(XLSX)

Table S6 **AR sequences and their positions in DisProt proteins.** UniProt and DisProt IDs and the AR/LCR percentages in respective proteins are given.
(XLSX)

## References

[1] Tompa P (2003) Intrinsically Unstructured Proteins Evolve by Repeat Expansion. BioEssays 25: 847–855. 12938174 10.1002/bies.10324

[2] Radivojac P, Iakoucheva LM, Oldfield CJ, Obradovic Z, Uversky VN, et al (2007) Intrinsic Disorder and Functional Proteomics. Biophys J 92: 1439–1456. 17158572 10.1529/biophysj.106.094045 PMC1796814

[3] Dosztányi Z, Mészáros B, Simon I (2010) Bioinformatical Approaches to Characterize Intrinsically Disordered/Unstructured Proteins. Brief Bioinform 11: 225–243. 20007729 10.1093/bib/bbp061

[4] Sickmeier M, Hamilton JA, LeGall T, Vacic V, Cortese MS, et al (2007) Disprot: The Database of Disordered Proteins. Nuc Acids Res 35: D786–D793. 10.1093/nar/gkl893 PMC1751543 17145717

[5] Bryson K, Cozzetto D, Jones DT (2007) Computer-Assisted Protein Domain Boundary Prediction Using the Dom-Pred Server. Curr Protein Pept Sci 8: 181–188. 17430199 10.2174/138920307780363415

[6] Dunker A, Lawson J, Brown C, Williams R, Romero P, et al (2001) Intrinsically Disordered Protein. J Mol Graph Model 19: 26–59. 11381529 10.1016/s1093-3263(00)00138-8

[7] Pietrosemoli N, Garcia-Martin JA, Solano R, Pazos F (2013) Genome-Wide Analysis of Protein Disorder in Arabidopsis Thaliana: Implications for Plant Environmental Adaptation. PLOS ONE 8: e55524. 23408995 10.1371/journal.pone.0055524 PMC3567104

[8] Maestro B, Galan B, Alfonso C, Rivas G, Prieto MA, et al (2013) A New Family of Intrinsically Disordered Proteins: Structural Characterization of the Major Phasin PhaF from *Pseudomonas putida* KT2440. PLOS ONE 8: e56904. 23457638 10.1371/journal.pone.0056904 PMC3574117

[9] Burra PV, Kalmar L, Tompa P (2010) Reduction in Structural Disorder and Functional Complexity in the Thermal Adaptation of Prokaryotes. PLOS ONE 5: e12069. 20711457 10.1371/journal.pone.0012069 PMC2920320

[10] Orosz F, Ovadi J (2011) Proteins without 3D Structure: Definition, Detection and Beyond. Bioinformatics 27: 1449–1454. 21493654 10.1093/bioinformatics/btr175

[11] Schweers O, Schoenbrunn-Hanebeck E, Marx A, Mandelkow E (1994) Structural Studies of Tau Protein and Alzheimer Paired Helical Filaments Show No Evidence for Beta-Structure. J Biol Chem 269: 24290–24297. 7929085

[12] Hsu LJ, Sagara Y, Arroyo A, Rockenstein E, Sisk A, et al (2000) α-Synuclein Promotes Mitochondrial Deficit and Oxidative Stress. Am J Pathol 157: 401–410. 10934145 10.1016/s0002-9440(10)64553-1 PMC1850140

[13] Uversky VN (2002) What Does It Mean to Be Natively Unfolded? Eur J Biochem 269: 2–12. 11784292 10.1046/j.0014-2956.2001.02649.x

[14] Ahmad A, Uversky VN, Hong DP, Fink AL (2005) Early Events in the Fibrillation of Monomeric Insulin. J Biol Chem 280: 42669–42675. 16246845 10.1074/jbc.M504298200

[15] Weinreb P, Zhen W, Poon A, Conway K, Lansbury P (1996) NACP, A Protein Implicated in Alzheimer's Disease and Learning, is Natively Unfolded. Biochemistry 35: 13709–13715. 8901511 10.1021/bi961799n

[16] Wright P, Dyson H (1999) Intrinsically Unstructured Proteins: Re-Assessing the Protein Structure-Function Paradigm. J Mol Biol 293: 321–331. 10550212 10.1006/jmbi.1999.3110

[17] Dunker A, Cortese M, Romero P, Iakoucheva L, Uversky V (2005) Flexible Nets: The Roles of Intrinsic Disorder in Protein Interaction Networks. FEBS J 272: 5129–5148. 16218947 10.1111/j.1742-4658.2005.04948.x

[18] Uversky VN (2003) Protein Folding Revisited. A Polypeptide Chain at the Folding-Misfolding-Nonfolding Cross-Roads: Which Way to Go? Cell Mol Life Sci 60: 1852–1871. 14523548 10.1007/s00018-003-3096-6 PMC11146068

[19] Dunker K, Obradovic Z (2002) The Protein Trinity: Importance of Intrinsic Disorder for Protein Function. Hum Genome News 12: 13–14.

[20] Dosztanyi Z, Chen J, Dunker A, Simon I, Tompa P (2006) Disorder and Sequence Repeats in Hub Proteins and Their Implications for Network Evolution. J Proteome Res 5: 2985–2995. 17081050 10.1021/pr060171o

[21] Tompa P (2002) Intrinsically Unstructured Proteins. Trends Biochem Sci 27: 527–533. 12368089 10.1016/s0968-0004(02)02169-2

[22] Dyson H, Wright P (2002) Coupling of Folding and Binding for Unstructured Proteins. Curr Opin Struct Biol 12: 54–60. 11839490 10.1016/s0959-440x(02)00289-0

[23] Uversky VN, Fink AL (2004) Conformational Constraints for Amyloid Fibrillation: The Importance of Being Unfolded. Biochim Biophys Acta Proteins Proteomics 1698: 131–153. 10.1016/j.bbapap.2003.12.008 15134647

[24] Hegyi H, Buday L, Tompa P (2009) Intrinsic Structural Disorder Confers Cellular Viability on Oncogenic Fusion Proteins. PLOS Comput Biol 5: e1000552. 19888473 10.1371/journal.pcbi.1000552 PMC2768585

[25] Rochet JC, Lansbury PT Jr (2000) Amyloid Fibrillogenesis: Themes and Variations. Curr Opin Struct Biol 10: 60–68. 10679462 10.1016/s0959-440x(99)00049-4

[26] He B, Wang K, Liu Y, Xue B, Uversky VN, et al (2009) Predicting Intrinsic Disorder in Proteins: An Overview. Cell Res 19: 929–949. 19597536 10.1038/cr.2009.87

[27] Kelly JW (1998) The Alternative Conformations of Amyloidogenic Proteins and Their Multi-Step Assembly Pathways. Curr Opin Struct Biol 8: 101–106. 9519302 10.1016/s0959-440x(98)80016-x

[28] Spillantini MG, Schmidt ML, Lee VM, Trojanowski JQ, Jakes R, et al (1997) Alpha-Synuclein in Lewy Bodies. Nature 388: 839–840. 9278044 10.1038/42166

[29] Yagi H, Takeuchi H, Ogawa S, Ito N, Sakane I, et al (2010) Isolation of Short Peptide Fragments From Alpha-Synuclein Fibril Core Identifies a Residue Important for Fibril Nucleation: A Possible Implication for Diagnostic Applications. Biochim Biophys Acta Proteins Proteomics 1804: 2077–2087. 10.1016/j.bbapap.2010.07.007 20637318

[30] Uversky VN, Eliezer D (2009) Biophysics of Parkinson's Disease: Structure and Aggregation of Alpha-Synuclein. Curr Protein Pept Sci 10: 483–499. 19538146 10.2174/138920309789351921 PMC3786709

[31] Fandrich M, Forge V, Buder K, Kittler M, Dobson C, et al (2003) Myoglobin Forms Amyloid Fibrils by Association of Unfolded Polypeptide Segments. Proc Natl Acad Sci U S A 100: 15463–15468. 14665689 10.1073/pnas.0303758100 PMC307590

[32] Goldschmidt L, Teng PK, Riek R, Eisenberg D (2010) Identifying the Amylome, Proteins Capable of Forming Amyloid-Like Fibrils. Proc Natl Acad Sci U S A 107: 3487–3492. 20133726 10.1073/pnas.0915166107 PMC2840437

[33] Ivanova M, Sawaya M, Gingery M, Attinger A, Eisenberg D (2004) An Amyloid-Forming Segment of β2-Microglobulin Suggests a Molecular Model for the Fibril. Proc Natl Acad Sci U S A 101: 10584–10589. 15249659 10.1073/pnas.0403756101 PMC489978

[34] Lopez de la Paz M, Serrano L (2004) Sequence Determinants of Amyloid Fibril Formation. Proc Natl Acad Sci U S A 101: 87–92. 14691246 10.1073/pnas.2634884100 PMC314143

[35] Esteras-Chopo A, Serrano L, de la Paz MLp (2005) The Amyloid Stretch Hypothesis: Recruiting Proteins toward the Dark Side. Proc Natl Acad Sci U S A 102: 16672–16677. 16263932 10.1073/pnas.0505905102 PMC1283810

[36] Teng PK, Eisenberg D (2009) Short Protein Segments Can Drive a Non-Fibrillizing Protein into the Amyloid State. Prot Eng Des Sel 22: 531–536. 10.1093/protein/gzp037 PMC2719503 19602569

[37] Dobson CM (1999) Protein Misfolding, Evolution and Disease. Trends Biochem Sci 24: 329–332. 10470028 10.1016/s0968-0004(99)01445-0

[38] von Bergen M, Friedhoff P, Biernat J, Heberle J, Mandelkow EM, et al (2000) Assembly of Tau Protein into Alzheimer Paired Helical Filaments Depends on a Local Sequence Motif (^306^VQIVYK^311^) Forming β Structure. Proc Natl Acad Sci U S A 97: 5129–5134. 10805776 10.1073/pnas.97.10.5129 PMC25793

[39] Thompson A, White AR, McLean C, Masters CL, Cappai R, et al (2000) Amyloidogenicity and Neurotoxicity of Peptides Corresponding to the Helical Regions of PrPC. Journal of Neurosci Res 62: 293–301. 11020222 10.1002/1097-4547(20001015)62:2<293::AID-JNR14>3.0.CO;2-Y

[40] Wootton JC (1994) Sequences with Unusual Amino Acid Compositions. Curr opin struct biol 4: 413–421.

[41] Wootton JC (1994) Non-Globular Domains in Protein Sequences: Automated Segmentation Using Complexity Measures. Comput Chem 18: 269–285. 7952898 10.1016/0097-8485(94)85023-2

[42] Wootton JC, Federhen S (1993) Statistics of Local Complexity in Amino Acid Sequences and Sequence Databases. Comput Chem 17: 149–163.

[43] Wang X, Zhang S, Zhang J, Huang X, Xu C, et al A Large Intrinsically Disordered Region in SKIP and Its Disorder-Order Transition Induced by PPIL1 Binding Revealed by NMR. J Biol Chem 285: 4951–4963. 20007319 10.1074/jbc.M109.087528 PMC2836099

[44] Pedersen JS, Christensen G, Otzen DE (2004) Modulation of S6 Fibrillation by Unfolding Rates and Gatekeeper Residues. J Mol Biol 341: 575–588. 15276845 10.1016/j.jmb.2004.06.020

[45] Schlessinger A, Punta M, Yachdav G (2009) Improved Disorder Prediction by Combination of Orthogonal Approaches. PLOS ONE 4: e4433. 19209228 10.1371/journal.pone.0004433 PMC2635965

[46] Lise S, Jones DT (2005) Sequence Patterns Associated with Disordered Regions in Proteins. Proteins 58: 144–150. 15476208 10.1002/prot.20279

[47] Romero P, Obradovic Z, Li X, Garner E, Brown C, et al (2001) Sequence Complexity of Disordered Protein. Proteins 42: 38–48. 11093259 10.1002/1097-0134(20010101)42:1<38::aid-prot50>3.0.co;2-3

[48] Danzer KM, Ruf WP, Putcha P, Joyner D, Hashimoto T, et al Heat-Shock Protein 70 Modulates Toxic Extracellular α-Synuclein Oligomers and Rescues Trans-Synaptic Toxicity. FASEB J 25: 326–336. 20876215 10.1096/fj.10-164624 PMC3005424

[49] Huntley M, Golding G (2002) Simple Sequences Are Rare in the Protein Data Bank. Proteins 48: 134–140. 12012345 10.1002/prot.10150

[50] Magrane M, Consortium U (2011) UniProt Knowledgebase: A Hub of Integrated Protein Data. Database 2011: bar009. 21447597 10.1093/database/bar009 PMC3070428

[51] Fukuchi S, Sakamoto S, Nobe Y, Murakami SD, Amemiya T, et al (2012) IDEAL: Intrinsically Disordered Proteins with Extensive Annotations and Literature. Nuc Acids Res 40: D507–D511. 10.1093/nar/gkr884 PMC3245138 22067451

[52] Bemporad F (2006) Sequence and Structural Determinants of Amyloid Fibril Formation. Acc Chem Res 39: 620–627. 16981678 10.1021/ar050067x

[53] Caflisch A (2006) Computational Models for the Prediction of Polypeptide Aggregation Propensity. Curr Opin Chem Biol 10: 437–444. 16880001 10.1016/j.cbpa.2006.07.009

[54] Chiti F, Dobson C (2006) Protein Misfolding, Functional Amyloid, and Human Disease. Annu Rev Biochem 75: 333–366. 16756495 10.1146/annurev.biochem.75.101304.123901

[55] Fernandez-Escamilla A, Rousseau F, Schymkowitz J, Serrano L (2004) Prediction of Sequence-Dependent and Mutational Effects on the Aggregation of Peptides and Proteins. Nat Biotechnol 22: 1302–11306. 15361882 10.1038/nbt1012

[56] Maurer-Stroh S, Debulpaep M, Kuemmerer N, de la Paz ML, Martins IC, et al (2010) Exploring the Sequence Determinants of Amyloid Structure Using Position-Specific Scoring Matrices. Nat Meth 7: 237–242. 10.1038/nmeth.1432 20154676

[57] Shin SW, Kim SM (2005) A New Algorithm for Detecting Low-Complexity Regions in Protein Sequences. Bioinformatics 21: 160–170. 15333459 10.1093/bioinformatics/bth497

[58] Albà MM, Laskowski RA, Hancock JM (2002) Detecting Cryptically Simple Protein Sequences Using the SIMPLE Algorithm. Bioinformatics 18: 672–678. 12050063 10.1093/bioinformatics/18.5.672

[59] Raghava G (2000) APSSP2: Protein Secondary Structure Prediction Using Nearest Neighbor and Neural Network Approach. CASP 4: 75–76.

[60] Jones D (1999) Protein secondary Structure Prediction Based on Position-Specific Scoring Matrices. J Mol Biol 292: 195–202. 10493868 10.1006/jmbi.1999.3091

[61] Dosztanyi Z, Csizmok V, Tompa P, Simon I (2005) IUPred: Web Server for the Pre-Diction of Intrinsically Unstructured Regions of Proteins Based on Estimated Energy Content. Bioinformatics 21: 3433–3434. 15955779 10.1093/bioinformatics/bti541

[62] Schultz Jr, Milpetz F, Bork P, Ponting CP (1998) SMART, A Simple Modular Architecture Research Tool: Identification of Signaling Domains. Proc Natl Acad Sci 95: 5857–5864. 9600884 10.1073/pnas.95.11.5857 PMC34487

[63] Schad E, Kalmar L, Tompa P (2013) Exon-Phase Symmetry and Intrinsic Structural Disorder Promote Modular Evolution in the Human Genome. Nuc Acids Res 41: 4409–4422. 10.1093/nar/gkt110 PMC3632108 23460204

[64] Frousios KK, Iconomidou VA, Karletidi CM, Hamodrakas SJ (2009) Amyloidogenic Determinants Are Usually Not Buried. BMC Struct Biol 9: 9. 19589171 10.1186/1472-6807-9-44 PMC2714319

[65] Tian J, Wu NF, Guo J, Fan YL (2009) Prediction of Amyloid Fibril-Forming Segments Based on a Support Vector Machine. BMC Bioinformatics 10: 8. 19208147 10.1186/1471-2105-10-S1-S45 PMC2648769

[66] Garbuzynskiy SO, Lobanov MY, Galzitskaya OV (2010) FoldAmyloid: A Method of Prediction of Amyloidogenic Regions from Protein Sequence. Bioinformatics 26: 326–332. 20019059 10.1093/bioinformatics/btp691

[67] Yoon S, Welsh WJ (2004) Detecting Hidden Sequence Propensity for Amyloid Fibril Formation. Protein Sci 13: 2149–2160. 15273309 10.1110/ps.04790604 PMC2279810

[68] Tartaglia GG, Cavalli A, Pellarin R, Caflisch A (2005) Prediction of Aggregation Rate and Aggregation-Prone Segments in Polypeptide Sequences. Protein Sci 14: 2723–2734. 16195556 10.1110/ps.051471205 PMC2253302

[69] Pawar AP, DuBay KF, Zurdo J, Chiti F, Vendruscolo M, et al (2005) Prediction of “Aggregation-Prone” and “Aggregation-Susceptible” Regions in Proteins Associated with Neurodegenerative Diseases. J Mol Biol 350: 379–392. 15925383 10.1016/j.jmb.2005.04.016

[70] Thompson M, Sievers S, Karanicolas J, Ivanova M, Baker D, et al (2006) The 3D Profile Method for Identifying Fibril-Forming Segments of Proteins. Proc Natl Acad Sci U S A 103: 4074–4078. 16537487 10.1073/pnas.0511295103 PMC1449648

[71] Conchillo-Sole O, de Groot NS, Aviles FX, Vendrell J, Daura X, et al (2007) AGGRESCAN: A Server for the Prediction and Evaluation of “Hot Spots” Of Aggregation in Polypeptides. BMC Bioinformatics 8: 17. 17324296 10.1186/1471-2105-8-65 PMC1828741

[72] Zhang Z, Chen H, Lai L (2007) Identification of Amyloid Fibril-Forming Segments Based on Structure and Residue-Based Statistical Potential. Bioinformatics 23: 2218–2225. 17599928 10.1093/bioinformatics/btm325

[73] Tartaglia GG, Pawar AP, Campioni S, Dobson CM, Chiti F, et al (2008) Prediction of Aggregation-Prone Regions in Structured Proteins. J Mol Biol 380: 425–436. 18514226 10.1016/j.jmb.2008.05.013

[74] Galzitskaya OV, Garbuzynskiy SO, Lobanov MY (2007) Expected Packing Density Allows Prediction of Both Amyloidogenic and Disordered Regions in Protein Chains. J Phys Condens Matter 19: 285225.

[75] Linding R, Schymkowitz J, Rousseau F, Diella F, Serrano L (2004) A Comparative Study of the Relationship Between Protein Structure and β-Aggregation in Globular and Intrinsically Disordered Proteins. J Mol Biol 342: 345–353. 15313629 10.1016/j.jmb.2004.06.088

[76] Calamai M, Chiti F, Dobson CM (2005) Amyloid Fibril Formation Can Proceed from Different Conformations of a Partially Unfolded Protein. Biophys J 89: 4201–4210. 16169975 10.1529/biophysj.105.068726 PMC1366985

[77] Monsellier E, Ramazzotti M, Taddei N, Chiti F (2008) Aggregation Propensity of the Human Proteome. PLOS Comput Biol 4: 9. 10.1371/journal.pcbi.1000199 PMC2557143 18927604

[78] Huang K, Maiti NC, Phillips NB, Carey PR, Weiss MA (2006) Structure-Specific Effects of Protein Topology on Cross-β Assembly: Studies of Insulin Fibrillation. Biochemistry 45: 10278–10293. 16922503 10.1021/bi060879g

[79] Mohan A, Oldfield CJ, Radivojac P, Vacic V, Cortese MS, et al (2006) Analysis of Molecular Recognition Features (MoRFs). J Mol Biol 362: 1043–1059. 16935303 10.1016/j.jmb.2006.07.087

[80] Vacic V, Oldfield CJ, Mohan A, Radivojac P, Cortese MS, et al (2007) Characterization of Molecular Recognition Features, MoRFs, and Their Binding Partners. J Proteome Res 6: 2351–2366. 17488107 10.1021/pr0701411 PMC2570643

[81] Disfani FM, Hsu WL, Mizianty MJ, Oldfield CJ, Xue B, et al (2012) MoRFpred, a Computational Tool for Sequence-based Prediction and Characterization of Short Disorder-to-order Transitioning Binding Regions in Proteins. Bioinformatics 28: I75–I83. 22689782 10.1093/bioinformatics/bts209 PMC3371841

[82] Dosztanyi Z, Meszaros B, Simon I (2009) ANCHOR: Web Server for Predicting Protein Binding Regions in Disordered Proteins. Bioinformatics 25: 2745–2746. 19717576 10.1093/bioinformatics/btp518 PMC2759549

[83] Karlin S, Burge C (1996) Trinucleotide Repeats and Long Homopeptides in Genes and Proteins Associated with Nervous System Disease and Development. Proc Natl Acad Sci U S A 93: 1560–1565. 8643671 10.1073/pnas.93.4.1560 PMC39980

[84] Al-Ali H, Rieger ME, Seldeen KL, Harris TK, Farooq A, et al (2010) Biophysical Characterization Reveals Structural Disorder in the Developmental Transcriptional Regulator LBH. Biochem Biophys Res Commun 391: 1104–1109. 20005203 10.1016/j.bbrc.2009.12.032 PMC2827303

[85] Angarica VE, Ventura S, Sancho J (2013) Discovering Putative Prion Sequences in Complete Proteomes Using Probabilistic Representations of Q/N-rich Domains. BMC Genomics 14: 316. 23663289 10.1186/1471-2164-14-316 PMC3654983

[86] Du Z (2011) The Complexity and Implications of Yeast Prion Domains. Prion 5: 311–316. 22156731 10.4161/pri.5.4.18304 PMC4012399

[87] Halfmann R, Alberti S, Krishnan R, Lyle N, O'Donnell CW, et al (2011) Opposing Effects of Glutamine and Asparagine Govern Prion Formation by Intrinsically Disordered Proteins. Mol Cell 43: 72–84. 21726811 10.1016/j.molcel.2011.05.013 PMC3132398

[88] Malinovska L, Kroschwald S, Alberti S (2013) Protein Disorder, Prion Propensities, and Self-Organizing Macromolecular Collectives. Biochim Biophys Acta Proteins Proteomics 1834: 918–931. 10.1016/j.bbapap.2013.01.003 23328411

[89] Mohan A, Uversky VN, Radivojac P (2009) Influence of Sequence Changes and Environment on Intrinsically Disordered Proteins. PLOS Comput Biol 5: e1000497. 19730682 10.1371/journal.pcbi.1000497 PMC2727479

