# Supplementary material for: Sequence Complexity of Amyloidogenic Regions in Intrinsically Disordered Human Proteins
Source: PLoS One. 2014 Mar 3;9(3):e89781. doi: 10.1371/journal.pone.0089781 (PMC3940659; doi:10.1371/journal.pone.0089781)
Supplement: Text S1 — Stable distribution function. Details of the statistical distribution function applied to AR/LCR length/content distribution. (DOCX) [file pone.0089781.s001.docx]

Stable distribution

- A stable distribution is defined in terms of its characteristic function $\phi(t)$, which satisfies a functional equation where for any *a* and *b* there exist *c* and *h* such that $\phi\left( a t \right) \phi\left( b t \right)=\phi\left( c t \right)exp(iht)$. The general solution to the functional equation has four parameters
- StableDistribution allows 0<α≤2, -1<β≤1, μ to be any real number, and σ to be any positive real number
- CharacteristicFunction[StableDistribution[0, α, ...], t] is continuous in α and given by

$$\left\{ \begin{matrix} \exp\left( i\mu t-\sigma\left| t \right|(1+2i \beta/\pi sgn(t)log(\left| t\sigma\right|)) \right) \alpha=1 \\ \exp\left( i\mu t-\left| t\sigma\right|^{\alpha}\left( 1-i\beta\tan\left( \frac{\pi\alpha}{2} \right)sgn\left( t \right)(\left| t\sigma\right|^{1-\alpha}-1) \right) \right) \alpha\neq1 \end{matrix} \right.$$

- CharacteristicFunction[StableDistribution[1, α, ...], t] is discontinuous in α and given by

$$\left\{ \begin{matrix} \exp\left( i\mu t-\sigma\left| t \right|(1+2i \beta/\pi sgn(t)log(\left| t \right|)) \right) \alpha=1 \\ \exp\left( i\mu t-\left| t\sigma\right|^{\alpha}\left( 1-i\beta\tan\left( \frac{\pi\alpha}{2} \right)sgn\left( t \right) \right) \right) \alpha\neq1 \end{matrix} \right.$$
